# Personalized Cognitive Support via Social Robots

**DOI:** 10.3390/s25030888

**Published:** 2025-01-31

**Authors:** Jaime Andres Rincon Arango, Cedric Marco-Detchart, Vicente Javier Julian Inglada

**Affiliations:** 1 Departamento de Digitalización, Escuela Politécnica Superior, Universidad de Burgos, Ctra. Orón, 28, 09200 Miranda de Ebro, Spain; 2 Departamento de Estadistica, Informatica y Matematicas, Universidad Publica de Navarra, Campus Arrosadia, 31006 Pamplona, Spain; cedric.marco@unavarra.es; 3 Valencian Research Institute for Artificial Intelligence, Universitat Politècnica de València, 46022 Valencia, Spain; vjulian@upv.es; 4 Valencian Graduate School and Research Network of Artificial Intelligence (VALGRAI), Universitat Politècnica de València, Camí de Vera s/n, 46022 Valencia, Spain

**Keywords:** cognitive assistant, wearable, emotion detection, signal processing, elderly well-being

## Abstract

This paper explores the use of personalized cognitive support through social robots to assist the elderly in maintaining cognitive health and emotional well-being. As aging populations grow, the demand for innovative solutions to address issues like loneliness, cognitive decline, and physical limitations increases. The studied social robots utilize machine learning and advanced sensor technology to deliver real-time adaptive interactions, including cognitive exercises, daily task assistance, and emotional support. Through responsive and personalized features, the robot enhances user autonomy and improves quality of life by monitoring physical and emotional states and adapting to the needs of each user. This study also examines the challenges of implementing assistive robots in home and healthcare settings, offering insights into the evolving role of AI-powered social robots in eldercare.

## 1. Introduction

Nowadays, social robots are emerging as promising tools for providing personalized cognitive support to individuals with diverse needs. These robots, designed to interact naturally and empathetically with users, can assist with daily tasks [1], offer reminders, and engage in cognitive stimulation activities [2].

The primary goal of these systems is to tailor their interactions to the individual characteristics and preferences of each user. This is achieved through the use of advanced machine learning algorithms and user modeling techniques. For instance, initiatives like the UPA4SAR project (http://www.upa4sar.unina.it/home, accessed on 8 November 2024) have shown that robots can deliver personalized care in the home for patients with mild cognitive impairment, adapting their behaviors and tasks based on the user’s changing needs and the environment [3].

These social robots leverage a combination of machine learning algorithms [4] and user modeling techniques to adapt to individual needs. Some of them employ a service-oriented approach to customize and adjust assistive tasks based on the user’s characteristics and the dynamic conditions of the environment. This includes the use of sensors to monitor the user’s state and modify the robot’s interactions in real time, ensuring a responsive and adaptive experience [5].

The use of such robots offers a range of benefits, directly contributing to the well-being of the patient. One of the most notable advantages is personalization, as these robots can tailor their actions to the specific needs of each individual, thereby enhancing the effectiveness of interventions. Another key benefit is the promotion of user autonomy; by assisting with daily tasks, the robots enable users to maintain their independence, reducing the burden on caregivers. In addition, accessibility is a crucial factor, as these robots can provide continuous support at home, which is particularly valuable for individuals with limited mobility.

Incorporating social robots into healthcare settings offers a promising solution to provide ongoing and tailored support, helping to improve both the quality of life of patients and the efficiency of care delivery. However, in addition to the benefits, these technologies also face a series of challenges that must be addressed gradually. One of the most significant challenges is the cost of these assistive robots, as their implementation can be expensive, limiting accessibility for many individuals. Another critical challenge is the acceptance of the user and caregiver, which can vary and is essential to the success of the intervention. Lastly, there is the issue of security and privacy. It is crucial to ensure that the personal and health data of users are protected, as these robots often rely on sensitive information to function effectively. Addressing these challenges is key to realizing the full potential of social robots in providing personalized, effective, and secure support.

Thanks to recent advances in artificial intelligence (AI), the personalization of healthcare through robotics [6] has progressed significantly. With cutting-edge technologies such as machine learning and advanced sensors, assistive robots can now learn and respond in real time to users’ health conditions, preferences, and behaviors. This offers a far more personalized and effective approach compared to traditional healthcare methods.

These modern assistive robotic systems can integrate sensors and monitoring devices, such as smart wristbands or vital sign tracking systems, to collect data on the patient’s physical condition [7] (e.g., heart rate, blood pressure, physical activity). These data enable the robot to adjust its interactions, such as medication reminders or exercise routines, based on the patient’s current state, thus improving the accuracy and responsiveness of the care provided.

An important aspect to consider is the robot’s ability to adapt its behavior based on the patient’s emotional responses, energy levels, or overall behavior. For example, if a patient shows signs of fatigue, the robot can reduce the intensity of activities or offer emotional support through calming conversation, adjusting to the individual’s emotions and mental state.

Assistive robots can also help with daily tasks, such as dressing, eating, or mobility, tailored to the patient’s capabilities and limitations [8]. By customizing assistance according to the specific abilities of each person, these robots promote greater autonomy, allowing patients to maintain their independence for as long as possible. This level of personalization ensures that the support provided is both effective and respectful of the patient’s needs and preferences.

Many elderly individuals experience some form of cognitive decline or neurodegenerative diseases, such as Alzheimer’s. In these cases, robots can personalize cognitive activities, such as memory games, based on the progression of the disease. By adjusting the difficulty and content of these cognitive exercises, robots can help maintain cognitive function while adapting to the patient’s changing mental abilities. This tailored approach ensures that the activities remain engaging and effective, offering support that evolves with the patient’s condition, which is crucial in promoting mental well-being and slowing cognitive decline.

Considering all of this, this paper contributes to the field by analyzing and presenting various illustrative examples of cognitive assistant technologies, each selected to showcase the versatility and breadth of potential applications within this rapidly evolving area. Through these examples, we aim to highlight the expanding capabilities of cognitive systems and their practical implementation in real-world settings. This study underscores the synergistic role of AI and robotic platforms, particularly in enhancing human–computer interaction and improving the quality of life for users, thus offering a holistic view of the potential impact of cognitive assistants in diverse environments.

Cognitive systems are advanced technologies designed to emulate and enhance human cognitive capabilities, enabling more natural and efficient interactions between humans and machines. To achieve this, they must incorporate a set of essential capabilities that effectively replicate human cognitive functions [9].

One of the most prominent capabilities is perception and sensory processing, which allows cognitive systems to interpret data from multiple sources, such as images, sounds, and texts. This facilitates the recognition of patterns, objects, and emotions. For instance, in the healthcare sector, these systems are widely used to analyze medical images and detect anomalies with high precision [10].

A fundamental component of these systems is attention to and prioritization of relevant information. In data-rich environments, cognitive systems filter and select critical information, improving efficiency in tasks such as security monitoring or social media analysis to identify trends and alerts. This is complemented by memory and data storage, enabling the recording of large volumes of data and facilitating their retrieval. This capability is particularly valuable in sectors like finance and education, where quick access to historical data is crucial for decision-making [11,12].

The ability to learn and adapt is arguably one of the most significant features. By leveraging machine learning algorithms, these systems adjust to new data and improve their performance over time. This makes them essential for applications like virtual assistants, which personalize responses based on user preferences and interaction history [13].

Furthermore, by integrating perception, storage, and learning capabilities, cognitive systems must include advanced modules for reasoning and decision-making. These modules process complex information to generate recommendations and make autonomous decisions, with applications ranging from medical diagnostic systems to the efficient management of supply chains [14].

To complement these features, an advanced language and communication system is indispensable. Such systems enable cognitive systems to process and generate natural language, facilitating more human-like interactions in applications such as chatbots, automated translation, and customer service systems [15].

The implementation of these capabilities in real-world applications has revolutionized sectors such as education, healthcare, finance, and entertainment, enhancing both operational efficiency and user experience. However, the development and deployment of these systems require a robust ethical framework and continuous evaluation to ensure alignment with human values, fairness, and privacy.

The rest of this paper is structured as follows: Section 2 reviews related work, providing an overview of existing cognitive assistant technologies and their applications in eldercare and other domains. Section 3 presents some of our developed approaches, along with other existing proposals, detailing the design and functionality of social robots equipped with cognitive support capabilities. Then, in Section 4, the actual limitations of social robotics are analyzed, and in Section 5, the main ethical concerns about assistant technologies are discussed. Finally, Section 6 concludes the paper, summarizing the main contributions and outlining potential directions for future research in advancing cognitive support technologies.

## 2. Related Work

The global phenomenon of an aging population presents significant public health challenges. Rehabilitation plays a crucial role in mitigating the disabilities and limitations that often accompany old age. Various studies highlight the importance of physical activities in reducing functional limitations among older adults, thereby improving their quality of life. Engaging in physical exercise not only enhances physical health but also contributes to overall well-being.

In addition to physical rehabilitation, companion robots play a significant role in elderly care, helping to mitigate issues related to anxiety and loneliness. These robots come in various forms, with robotic pets being particularly widespread. Notable examples include the seal-shaped robot Paro [16], the dog-shaped JfA (https://joyforall.com, accessed on 8 November 2024), the cat-shaped JfA [17], the dinosaur-shaped Pleo [18], and the dog-shaped AIBO [19]. Some companion robots are designed to resemble specific creatures, while others, like LOVOT [20], are intentionally designed not to resemble any specific animal. These companion robots have proven beneficial in providing emotional support and companionship to older adults, significantly improving their mental well-being. In [21], the authors present CARESSER, which is a novel framework for socially assistive robots that actively learns personalized therapeutic behaviors by combining therapists’ expertise (knowledge-driven) and demonstrations (data-driven). The approach enables fast, autonomous learning of patient-specific policies without complex reward functions. User studies with older adults showed that the robot effectively maintained patients’ performance during cognitive exercises and aligned its assistance with therapists’ preferences. Casper [22] is another example: it is a robot learning architecture that combines learning from demonstration (LfD) and reinforcement learning (RL) to teach socially assistive robots personalized behaviors. Experiments with the robot Casper in a tea-making task showed it effectively learned personalized behaviors. Related to the use of RL techniques, the work presented in [23] surveys RL approaches in social robotics, focusing on physically embodied robots and real-world human–robot interactions. It categorizes studies by RL methods, reward design, and communication media, highlighting challenges and opportunities in the field. The goal is to provide a comprehensive reference for researchers applying RL in social robotics.

Moreover, older adults need to engage in physical exercise, as it helps them feel better. The study conducted by Meghan H. McDonough et al. [24] developed a framework to distinguish social benefits, such as role models, social networks, social participation, social connection, and social support. This social benefit framework provides a practical tool for conceptualizing the social advantages that may be relevant in group physical activities for older adults.

Various studies highlight the pivotal role of robotic assistants in elderly care, emphasizing their impact on interaction and support for seniors. These robots address diverse issues, such as dementia, cognitive therapies, and social interaction, among others. For instance, Perugia et al. [25] introduced a novel tool to measure engagement in individuals with dementia, utilizing board games and interactions with the social robot Pleo. Similarly, Šabanović et al. [26] evaluated the PARO seal robot in a multisensory behavioral therapy context with elderly individuals experiencing varying levels of dementia. Their study demonstrates PARO’s efficacy in enhancing social interaction modalities, including visual, verbal, and physical interactions. Another example is Ryan [27], which is a social robot designed to support older adults with depression and dementia by integrating artificial emotional intelligence. The real study proposed by the authors shows that interactions with Ryan improved users’ moods, with the robot perceived as engaging and likable.

Some robots serve as companion robots, aiming to alleviate loneliness and isolation among seniors. MARIO [28], for example, is specifically designed to promote social connectivity and reduce loneliness among people living with dementia by providing access to a variety of interactive apps via voice commands or touchscreen interaction. In the same way, ElliQ [29] is a companion robot designed to assist older adults, promoting independence and well-being. It uses AI techniques to engage in personalized conversations, remind users to take medications, suggest physical and cognitive activities, and facilitate communication with family through video calls. Its design features a lamp-like structure and an interactive tablet, providing a user-friendly and accessible interface. Its strengths include a user-friendly design, tailored engagement, and health-focused support. However, it has limitations, such as a lack of mobility, reliance on Internet connectivity, high cost, and conversational depth that may not fully meet all users’ expectations. Its high price makes it inaccessible for many potential users, limiting its ability to fully address the needs of a broader audience.

In general, the use of cognitive assistants to help people with cognitive or physical disabilities offers significant potential to improve their daily lives, promote greater independence, and improve access to healthcare and educational resources. As the field of cognitive assistive technology evolves, it is crucial to prioritize responsible and ethical design and use. This approach will help maximize the benefits of cognitive assistants while mitigating potential risks and challenges.

## 3. Proposed Approaches

In this section, we illustrate a range of cognitive assistant robots, each designed to showcase the flexibility and potential applications of assistive technologies in eldercare. These examples demonstrate the expanding capabilities of AI-enhanced robotic systems and their practical implementations in real-world contexts. Through their unique functions, these robots exemplify the synergy between AI and robotics in advancing human–computer interaction.

Recent developments in social robotics have yielded numerous assistive robots tailored specifically for eldercare. While some of these robots have already reached advanced commercial stages, being integrated into homes and care centers, others are still in the early stages of research and development, exploring new functionalities and ways to improve the quality of life of this population. In this article, we will primarily focus on robots that are still in the research phase, analyzing the challenges and opportunities presented in their design and development.

The design and development of assistive robots for the elderly is experiencing significant growth, driven by the global aging population and the need to offer technological solutions that complement traditional care systems. These robots not only focus on providing physical assistance or performing daily tasks but also address one of the most pressing issues faced by older adults: unwanted loneliness. Through simulated social interactions, personalized reminders, and emotional companionship, these robots have the potential to offer continuous company and assistance, significantly improving the psychological well-being of users.

Assistive robots for elderly care address a broad spectrum of needs beyond physical assistance, including alleviating loneliness, managing cognitive exercises, and supporting emotional well-being. Through simulated social interactions, personalized reminders, and companionship, these robots can significantly enhance psychological and emotional health. This requires a highly sophisticated design, as robots must operate autonomously within domestic environments, respond dynamically to user needs, and provide reliable monitoring.

Robots are beginning to play a crucial role in enhancing the quality of life of older adults. Thanks to AI, robots can learn and adapt to the individual preferences of each user, offering an increasingly personalized and efficient assistance experience. For example, through machine learning algorithms, robots can adjust their behavior according to the user’s emotional state or daily habits, offering specific recommendations or modifying their tone and interaction style based on the situation.

On the hardware side, advancements in motion sensors, cameras, and haptic devices allow robots to have better environmental perception and react more precisely and safely to the movements and actions of older adults.

The advancements in sensor technology, such as cameras that are increasingly compact and, in some cases, equipped with artificial intelligence capabilities, have significantly boosted the development of more efficient and versatile assistant robots. Additionally, these robots are equipped with a variety of other sensors, such as inertial and microwave sensors, enabling them to detect movements with high precision, further enhancing their functionality and adaptability in various environments.

In the domain of motion detection and non-invasive vital sign monitoring, mmWave (millimeter-wave) motion sensors stand out. These sensors utilize high-frequency electromagnetic waves to detect subtle movements and monitor heart rates without physical contact. Their ability to penetrate materials like plastic and textiles makes them ideal for applications in automotive environments and assisted care settings [30].

For computer vision applications, advanced depth cameras offer substantial advantages, with many supporting the integration of pre-trained models for edge detection. Devices such as Intel RealSense cameras provide detailed three-dimensional environmental data, allowing robots to map and navigate spaces with precision while recognizing human gestures and movements. Additionally, compact LiDAR sensors, such as those from Velodyne LiDAR, deliver high-resolution detection of objects and individuals in the robot’s immediate surroundings in a compact form factor [31].

For discreet monitoring, Wi-Fi Sensing systems leverage devices like Threshold Care’s Motion Wi-Fi Sensing plugs, which detect movements through Wi-Fi signal interference, enabling the monitoring of older adults without the need for cameras, thereby preserving privacy. Similarly, advanced infrared sensors, such as those used in the Ally Cares system, monitor movement patterns and activity, alerting caregivers to unusual behaviors in older adults [32].

The incorporation of health sensors is also critical, as it enables the continuous monitoring of vital signs such as heart rate or blood pressure, generating alerts if anomalies are detected that may require medical intervention.

Moreover, the implementation of these robots not only has individual benefits but also represents a solution at the social and economic levels. As the aging population grows, healthcare and caregiving systems are under pressure, and robots are expected to alleviate part of this burden by providing autonomous assistance, allowing human caregivers to focus on more complex or emotional tasks. By reducing the exclusive dependence on human caregivers, these devices also help maintain the autonomy of older adults, enabling them to live more independently in their homes for longer periods.

However, significant challenges remain. One of the most important is ensuring that these robots are not only technically efficient but also ethically responsible. The design of these robots must consider aspects related to privacy, as the collection of personal and health data is an integral part of their operation. Additionally, it is essential to avoid creating excessive emotional dependence on the robots, maintaining a clear distinction between interaction with a machine and human relationships.

In summary, assistive robots for older adults represent one of the most promising areas of robotics today, with significant potential to improve the quality of life for an increasingly vulnerable population. As AI and robotics technologies continue to evolve, these devices will likely become indispensable companions in the future, providing not only physical assistance but also emotional and social support to older adults.

### 3.1. Emir: Emotional Intelligent Robot

**Figure 1:**
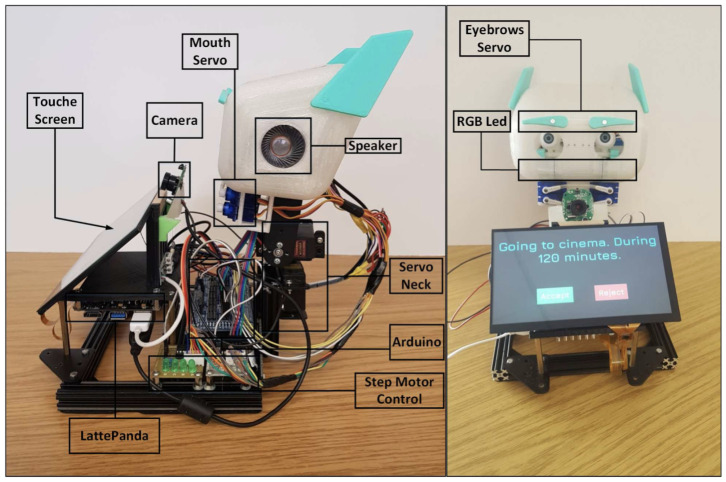
EmIR robot [33].

An example is the EmIR robot (Figure 1) (EMotional Intelligent Robot assistant) [33]. EmIR is an emotionally intelligent robot assistant designed to interact with users more naturally and efficiently by taking their emotional states into account. The emotional intelligence system integrated into EmIR allows it to recognize, interpret, and respond to human emotions using a combination of natural language processing techniques, facial expression analysis, and voice pattern monitoring.

The primary goal of EmIR is to provide personalized assistance, adjusting its behavior and responses based on the user’s emotional state, making it a valuable companion in areas such as customer service, education, and the care of elderly or disabled individuals. The robot’s ability to empathize with users enhances human–robot interaction, fostering trust and cooperation in various tasks.

EmIR’s technical capabilities include the integration of emotion recognition systems, dialogue planning, and real-time adaptation. EmIR can perform multiple functions, from offering recommendations to assisting with daily tasks, all while adjusting its interaction to maintain a positive emotional environment for the user.

The EmIR robot utilizes a convolutional neural network (CNN) for facial emotion detection, trained using the KDEF (Karolinska Directed Emotional Faces) dataset [34,35]. This dataset, which contains images of faces representing various emotions, serves as a robust foundation for training emotion recognition models.

The training process of the CNN with KDEF is conducted in several critical stages, each contributing to the system’s performance in emotion detection. In the initial stage, data preprocessing is performed, where the dataset images are resized and normalized to ensure consistency and improve training efficiency. Subsequently, the CNN architecture is designed, comprising multiple convolutional layers, followed by pooling layers, and concluding with fully connected layers that culminate in an output layer responsible for classifying the detected emotions.

During the training phase, the neural network processes the preprocessed images, adjusting its internal weights using optimization algorithms, such as gradient descent, to minimize the difference between the network’s predictions and the actual emotion labels. Finally, the model undergoes a validation and evaluation phase, where a subset of data is used to assess its performance and fine-tune hyperparameters. This step is critical to ensuring the model generalizes well to unseen data.

This structured approach enables EmIR to accurately identify and classify human emotions from facial images. This capability not only enhances the robot’s ability to interpret emotional cues but also facilitates more natural and effective interactions in various social environments.

However, other AI techniques, such as reinforcement learning, can be applied to address challenges in social robotics. In this context, Neziha Akalin et al. [23] present a comprehensive review of the application of such techniques in this field. This approach enables robots to enhance their ability to interact with humans by learning appropriate social behaviors through experience and environmental feedback. The article examines both traditional and advanced reinforcement learning methods, emphasizing the need to design algorithms that account for the inherent complexities of human interactions. This design ensures that robots can adapt to diverse social contexts and respond appropriately to the dynamics of these environments.

Additionally, the authors address the challenges associated with using reinforcement learning in real-world scenarios, such as the need for large volumes of training data and the difficulties in modeling complex human behaviors. As alternatives, solutions based on simulations and virtual environments are proposed, enabling robots to be effectively trained before deployment in real-world settings. In conclusion, this study highlights the potential of reinforcement learning to endow robots with advanced capabilities, facilitating their adaptation to complex social dynamics and improving their effectiveness and acceptance in everyday human interactions.

Building upon the framework of reinforcement learning, Antonio Andriella et al. [36] present a symbolic task-planning approach for cognitive robotic systems assisting individuals with mild dementia. It enables robots to adapt their behavior during brain-training exercises, offering personalized verbal and gestural support based on user performance. A safety module ensures physical interaction monitoring, enhancing usability. The study explores concepts such as proxemics and social force models, which enable robots to predict and adapt to human dynamics in shared environments. Furthermore, the authors review recent experiments that integrate social conventions into robotics, highlighting the necessity for robots to interpret and respond effectively to social cues. Collectively, the article underscores the potential of incorporating sociological principles to enhance the seamless integration of robots into human environments, fostering more harmonious and efficient interactions.

In the field of assistive robotics, it is essential to consider not only emotional and cognitive factors but also cultural aspects that influence human interactions. In this context, Birgit Lugrin et al. [37] present a hybrid approach to developing a culturally specific model of nonverbal behavior for anthropomorphic interfaces, such as virtual agents or humanoid robots. Recognizing that human behavior is deeply shaped by cultural context, the authors propose a Bayesian network-based model that integrates these cultural dependencies to simulate credible and socially acceptable human-like behaviors in robots. This study highlights the importance of incorporating cultural context into the design of robot behaviors as a means to enhance the quality and effectiveness of human–robot interaction in assistive and social environments.

In another way, as technologies advance, devices like companion robots are becoming increasingly complex and smaller in size. The advent of 5G networks, robotics, and AI enhances the capabilities of these systems, enabling the creation of smaller yet more powerful robots. These robots can embed AI models and communicate with cloud services, improving their functionality.

### 3.2. E-Bot

**Figure 2:**
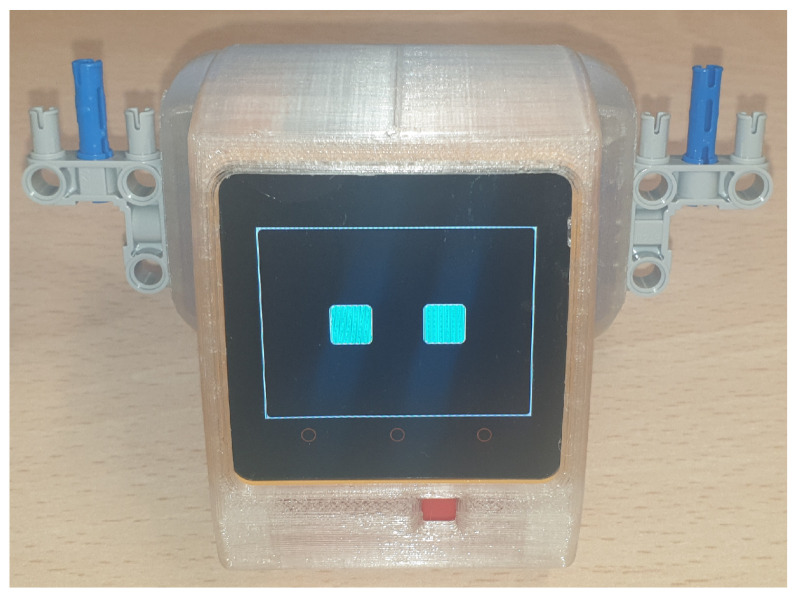
Real image of E-Bot companion robot [38].

In this case, a notable example is E-Bot [38] (Figure 2), which combines an ECG monitoring system with the Alexa voice assistant to facilitate user interaction. Users can issue voice commands, and the system captures physiological data, such as heart rate, via a chest strap equipped with the BMD101 cardiology chip. The collected data are sent to AWS for remote analysis by doctors or caregivers.

E-Bot is designed to be affordable, compact, and portable, making it suitable for home environments. It can be integrated with other monitoring devices (e.g., blood pressure monitors) and personalized via Alexa Skills. This customization enables enriched interactions and the creation of personalized exercise routines or health recommendations.

Additionally, the integration of TinyML algorithms enables the real-time analysis of ECG signals directly on the device (at the “Edge”). This allows E-Bot to classify heart signals as normal or abnormal, providing early detection of potential cardiac issues. The data are also securely sent to the cloud for further analysis and long-term storage.

E-Bot integrates a Deep Neural Network (DNN) designed to classify ECG signals as normal or abnormal. The model is trained using the PTB Diagnostic ECG dataset, allocating 80% of the data for training, 10% for testing, and 10% for validation. The network architecture consists of multiple fully connected layers, culminating in an output layer that produces binary classifications (normal or abnormal signals).

The training process employs the Adam optimizer, with a learning rate of 0.001, the binary cross-entropy loss function, 150 epochs, and a batch size of 32. Once training is completed, the model is deployed on an M5Stick-C Plus device (M5Stack Technology Co., Ltd., Shenzhen, China), enabling edge AI capabilities for the real-time classification of ECG signals.

The system achieves a classification accuracy of approximately 90%, validated through a confusion matrix. This level of accuracy highlights the robot’s ability to detect cardiac anomalies directly on local devices, minimizing latency while ensuring the efficient transmission of both raw and classified signals to AWS for further analysis.

### 3.3. MTY Robot

**Figure 3:**
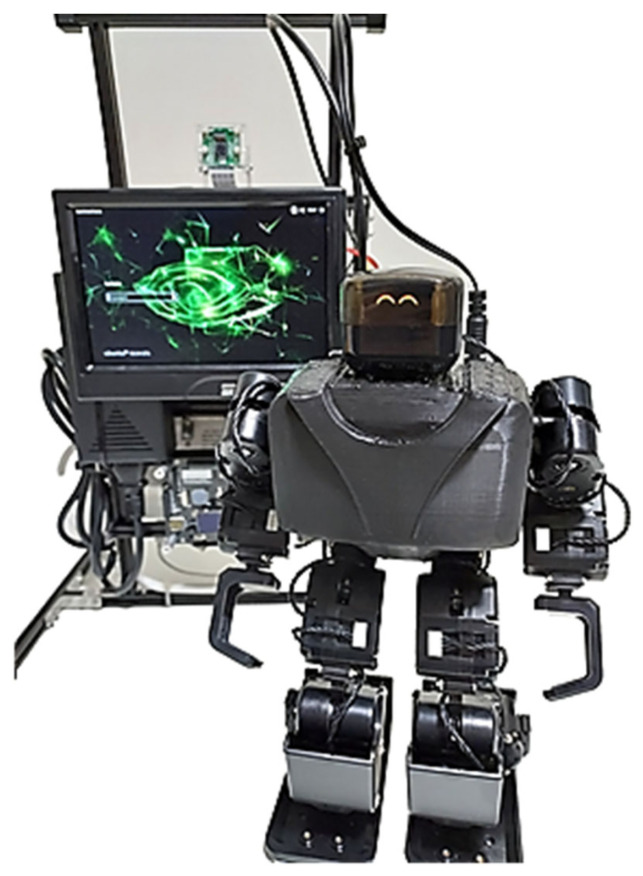
MTY robot [39].

Another important characteristic of social robots is user engagement, especially for older adults or individuals with disabilities. These robots can provide emotional and social support, promoting active interaction and improving users’ quality of life by encouraging their involvement in using the robot and in the social interactions it facilitates. An example of this type of robot is proposed in [39]. In this case, the authors propose an innovative social robot (see Figure 3) aimed at enhancing sustainable human interaction in home care services for aging populations, addressing issues of social isolation and loneliness among the elderly. The robot is designed to respond to users’ emotional needs through an advanced emotion recognition system, leveraging AI and neural network algorithms. With an empathetic interface and personalized responses, the robot has the potential to improve the mental and physical well-being of the elderly, helping to reduce their sense of loneliness. Key features of this robot include advanced emotional recognition, using machine learning techniques to interpret facial expressions in real time; personalized responses that allow for tailored interactions to support the user’s emotional well-being; and soft skills, incorporating empathetic interactions that enhance the quality of the user experience.

### 3.4. Assistant for Hand Rehabilitation

Robots can also serve as valuable tools in the rehabilitation of individuals, particularly by managing and encouraging the completion of controlled physical activities prescribed by rehabilitation specialists, such as physical therapists.

**Figure 4:**
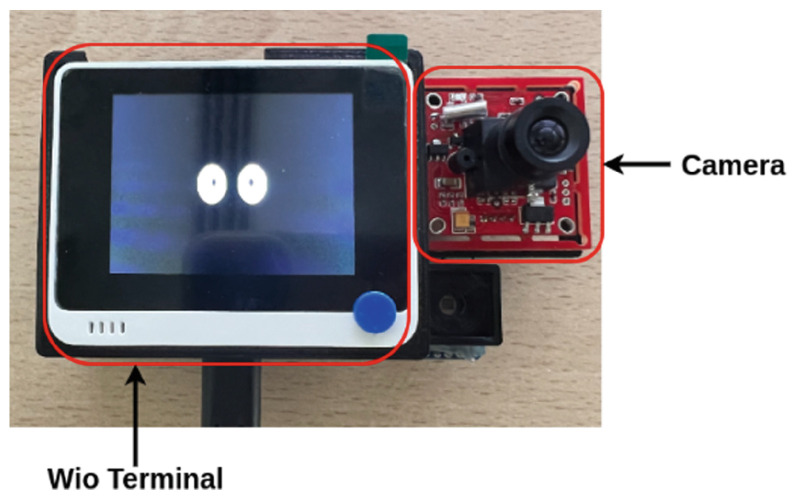
Prototype assistance [40].

An illustrative example of how assistive robots and cognitive assistants can support rehabilitation is presented by Rincon et al. [40], who introduce an affordable cognitive assistant robot designed specifically for the physical rehabilitation of older adults, focusing on hand exercises for arthritis patients. This robot is equipped with a camera to record exercises and an animated screen that provides instructions and feedback to the user (see Figure 4). Leveraging edge computing technology, the robot can operate offline with low latency, ensuring reliable functionality without relying on Internet connectivity. The system was evaluated with machine learning algorithms for gesture recognition, demonstrating strong results in accurately identifying and monitoring rehabilitation exercises.

This system leverages the MobileNet V1 architecture, a convolutional neural network specifically designed for edge devices, to classify rehabilitation exercises based on images captured by an integrated camera. The model was trained on a dataset of 1032 images, categorized into four exercise classes and an additional “no activity” class.

To optimize processing on the Wio Terminal, a device with limited memory and computational capacity, techniques such as resizing images to 32 × 32 pixels were applied. Key training hyperparameters included an Alpha value of 0.5, a dropout rate of 0.25, and a learning rate of 0.001.

The model’s performance was evaluated using a confusion matrix and the F1-Score, demonstrating high accuracy in exercise classification. This approach combines a cost-effective design and autonomous functionality in offline environments, providing an innovative solution for the monitoring and rehabilitation of older adults.

### 3.5. The Social Robot Mini

On the other hand, the social mini-robot explores the integration of Large Language Models (LLMs). The authors describe the design and implementation of the conversational agent, highlighting how LLMs enhance the robot’s ability to interact naturally and contextually with users. The study demonstrates that incorporating LLMs into social robots can significantly improve human–robot interaction, enabling more fluid and meaningful conversations.

**Figure 5:**
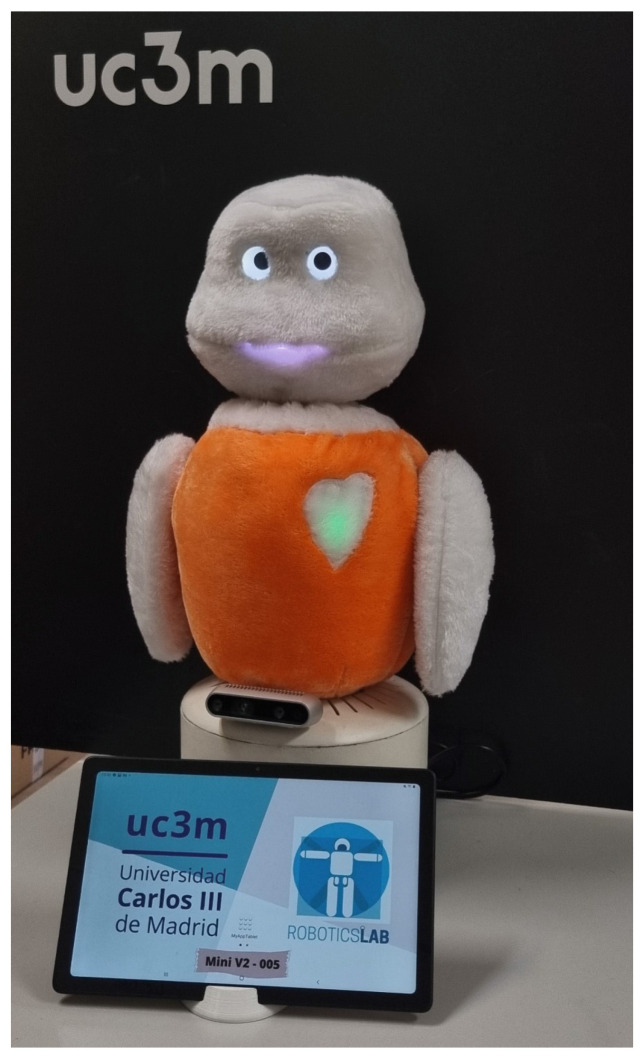
Social Robot uc3m [41].

The advent of LLMs has opened new doors for improving human–robot interactions, allowing users to communicate with robots more naturally, without relying on predefined conversations that previously made interactions feel artificial. An example of this integration is presented by Esteban-Lozano et al. [41], who incorporated a conversational agent based on an LLM, specifically GPT-3.5, into the social robot Mini (see Figure 5). This robot is designed to provide companionship and support for older adults in their daily lives. The system includes an automatic speech recognition (ASR) module and a text-to-speech (TTS) module, enabling smooth, bidirectional verbal interactions, particularly suited to older adults.

The findings suggest that conversational agents based on LLMs hold great potential for advancing the capabilities of social robots in various applications, such as education, healthcare, and customer service. This chapter contributes to the discourse on human-centered artificial intelligence and the development of AI systems that can seamlessly integrate into people’s daily activities.

The robot’s architecture features a decision-making system that selects interaction functionalities, called “skills”, in response to internal and external events. A perception system manages audio and video sensors, while the expression system controls actuators that allow the robot to make gestures and movements, giving it a more “lifelike” appearance. Its conversational ability is structured as a state machine with four phases: greeting, conversation, continuity, and farewell. This interaction flow can be initiated by either the robot or the user and continues until a predefined limit is reached, at which point the robot asks the user if they would like to continue. The prompt design was iterative, ensuring that the model maintained a friendly and approachable tone, specially tailored to interact with older adults; these prompts include blocks for context, instructions, and conversation topics.

The system was evaluated with 24 users who freely interacted with the robot. The results showed high usability, with a score of 78.5 on the System Usability Scale (SUS) and an average user interaction rating of 8.1 out of 10. Positive feedback highlighted the spontaneity and coherence in the robot’s responses, while noted drawbacks included occasional ASR errors and some delays in the robot’s responses.

These examples reflect the growing diversity and depth of cognitive assistant applications, each customized to meet specific user needs in various contexts. Whether enhancing commercial robots, providing emotionally aware recommendations, or supporting rehabilitation efforts, these systems demonstrate the transformative potential of cognitive assistants to augment human abilities and improve the quality of human–machine interactions. By seamlessly integrating intelligent decision-making, personalization, and social engagement, cognitive assistants are set to become an integral part of future technological ecosystems, playing crucial roles in fields such as healthcare, education, customer service, and personalized well-being.

With the inclusion of Large Language Models (LLMs) in assistive robotics, a powerful synergy emerges that further amplifies these capabilities. LLMs allow robots to interpret and generate natural language in advanced ways, enhancing communication and emotional interaction with users, particularly in eldercare scenarios. This integration could transform assistive robotics by enabling robots to understand not only users’ physical needs but also their emotional states and preferences, adapting to provide empathetic and contextually aware responses.

Through these illustrative cases, we gain valuable insight into the evolving landscape of cognitive assistants and intelligent agents. These systems are not only improving task efficiency but also reshaping the nature of social and emotional interactions between humans and machines. As cognitive assistants continue to evolve, their capacity to understand, adapt, and engage meaningfully will shape the future of assistive technologies, opening new horizons for human–computer collaboration.

The integration of AI with robotic platforms is significantly transforming human–computer interaction (HCI) by enabling more intuitive, adaptive, and efficient interactions between humans and machines [42]. This synergy combines AI’s advanced data processing and decision-making capabilities with robotics’ physical interaction skills, leading to key advancements in various domains.

One of the primary achievements lies in the ability of robots to establish natural and contextual interactions. Powered by AI, robots can process and generate natural language fluently, allowing them to understand ambiguous queries, provide relevant responses, and adapt their communication to the specific needs of each user. This not only enhances the user experience but also fosters a more effective and empathetic relationship between humans and robots.

### 3.6. LLMs in Cognitive Assistants and Assistive Robotics

In this subsection, we explore the transformative potential of Large Language Models (LLMs) in the realm of cognitive assistants. These technologies not only promise to enhance the ability of assistants to process and generate natural language but also open new possibilities for their application in assistive environments, particularly in robotics designed for elderly care.

We also highlight various national and European projects that are researching and developing technologies based on LLMs and assistive robotics. These initiatives aim to address the challenges associated with an aging population, promoting solutions that enhance the quality of life and autonomy of older adults. The integration of LLMs in this context allows robots to interact more empathetically and personally, adapting to the unique needs and preferences of each individual.

The use of LLMs in assistive robotics [43] is transforming how social robots interact with humans, endowing them with advanced capabilities to interpret, understand, and respond in complex contexts. Models like GPT have proven to be fundamental tools in the design of social robots, enabling them to process and generate natural language in a fluid and adaptive manner. These capabilities are critical for robots to interpret ambiguous questions, provide relevant information, and adjust their language to the specific needs and characteristics of users [44]. This is particularly important for older adults or individuals with cognitive disabilities, where clear and empathetic interaction is essential.

One of the most significant contributions of LLMs in this field is their ability to integrate user-specific data, such as preferences, interaction history, and personal characteristics, allowing them to deliver highly personalized responses and actions [45]. This level of personalization is particularly useful in assistive environments, where adaptability to individual needs is essential for ensuring the effectiveness and acceptance of robots.

In addition, LLMs exhibit continuous learning capabilities, enabling them to process large volumes of data and learn from new interactions. This allows social robots to dynamically adjust their behavior to changes in the environment or user needs, progressively improving their performance and adaptability. This constant learning ensures that robots can respond effectively to emerging demands, optimizing their utility in diverse scenarios.

Another significant advantage of LLMs lies in their integration with multimodal sensors, such as cameras, microphones, and haptic devices, which enhance their ability to interpret complex signals [46]. By combining visual, verbal, and tactile data, LLMs enable robots to identify emotions, intentions, and needs with greater accuracy. For instance, emotional recognition is enhanced when robots process facial expressions and tone of voice simultaneously, allowing them to tailor their interactions more appropriately and effectively.

Overall, large LLMs are redefining the potential of social robots in assistive robotics. Their ability to generate more natural, personalized, and adaptive interactions, combined with their integration with multimodal technologies, not only improves their technical capabilities but also significantly elevates the quality of the user experience [47]. This positions LLMs as key tools in the development of more effective, empathetic, and suitable assistive robots for the complex needs of human care.

Personalization plays a central role in this integration [48]. By incorporating user-specific data such as preferences, interaction history, and individual characteristics, AI enables robots to deliver highly customized responses and actions. This adaptability is particularly critical in assistive environments, where addressing individual needs increases the efficacy and acceptance of robotic assistance.

Another exciting challenge is equipping robots with continuous learning capabilities [49]. This approach allows robots to learn from real-time interactions, dynamically adjusting their behavior to changes in user behavior or the environment [50]. This iterative learning process not only improves the robots’ performance but also ensures more responsive and effective interactions over time.

The combination of AI with multimodal sensors, such as cameras, microphones, and haptic devices, significantly enhances robots’ capabilities [51]. AI can process visual, auditory, and tactile data simultaneously, enabling a deeper understanding of human emotions, intentions, and needs. For instance, by analyzing data from multiple sources, robots can more accurately interpret user emotions and adjust their interactions accordingly.

For instance, the GrowMeUp (https://cordis.europa.eu/project/id/643647, accessed on 31 January 2018) project demonstrated a 25% improvement in daily task independence among older adults over a three-month trial period, emphasizing the value of adaptable robotic assistants in promoting user autonomy. Similarly, the Mario project focused on addressing loneliness among dementia patients through interactive applications and observed a 40% increase in perceived emotional well-being during its pilot studies [28]. These outcomes underscore the importance of personalization and interaction in improving the quality of life for elderly users.

The RAMCIP project targeted patients with mild cognitive impairment and achieved a 30% increase in task independence, particularly in activities of daily living, through intelligent robotic assistance (https://cordis.europa.eu/project/id/643433, accessed on 30 June 2018). Meanwhile, MoveCare integrated cognitive and physical monitoring with AI-powered robots, enhancing adherence to prescribed cognitive and physical activities in elderly participants [52]. These results align with the goals of robots like EmIR and E-Bot, which aim to support cognitive health while fostering emotional well-being.

Furthermore, the SHAPES project explored scalable solutions for aging populations by integrating robotics and IoT technologies. Its findings highlighted the economic feasibility of modular robotic designs, which not only improve access but also reduce costs for end-users (https://cordis.europa.eu/project/id/857159, accessed on 31 December 2023). Finally, the SPRING project, focused on rehabilitation, reported a 15–20% improvement in adherence rates for physical therapy among participants, demonstrating the potential of robots to complement traditional healthcare approaches (https://cordis.europa.eu/project/id/871245, accessed on 31 May 2024).

Together, these findings illustrate the potential of social robots to deliver measurable improvements in autonomy, emotional health, and physical engagement. The evidence strongly supports the inclusion of cognitive and emotional personalization as central features in robotic systems like the proposed EmIR and E-Bot.

## 4. Current Limitations

Assistive robotics for elderly care faces several limitations that impact its effectiveness and acceptance in real-world applications. Among the primary technological challenges is the inability of robots to interpret and respond adequately to the complexities inherent in human interactions, particularly in situations requiring empathy and contextual understanding. Moreover, robots struggle to adapt to unstructured environments, such as domestic spaces, which present unpredictable obstacles and dynamic changes that are naturally manageable by humans [53].

Another critical challenge lies in the ethical implications of robot-assisted care. There is growing concern about the potential replacement of human caregivers, which could compromise both the quality of care and the emotional warmth essential for supporting elderly individuals. Additionally, the acceptance of robots by users remains inconsistent, influenced by factors such as familiarity with technology, personal preferences, and perceptions of the robots’ utility and reliability. These factors underscore the need to design robotic solutions that are culturally sensitive and adaptable to individual needs [54].

In terms of privacy and security, robotic systems pose significant concerns. Being continuously connected to the Internet, these devices collect and manage sensitive data, raising issues about protecting users’ privacy. Furthermore, their constant connectivity makes them vulnerable to cyberattacks, potentially resulting in physical and emotional harm to the elderly individuals who rely on them [55].

Economically, the high costs associated with the development, implementation, and maintenance of assistive robots present a major barrier to widespread adoption. In their current state, these technologies tend to benefit only those who can afford the necessary investment, thereby exacerbating inequalities in access and availability.

Lastly, current robots lack the skills required to fully meet the demands of elderly care, particularly for tasks that involve human judgment and adaptability. Their limited ability to tailor care to the individual needs of elderly users significantly reduces the effectiveness of the assistance provided [56].

Addressing these limitations is essential for achieving the effective integration of robotics into elderly care. This requires ensuring that these technologies complement, rather than replace, human care while respecting the rights, dignity, and specific needs of individuals [57]. Ethical, secure, accessible, and personalized assistive robotics will be crucial in addressing the challenges posed by an aging global population soon.

## 5. Ethical Considerations in Social Robotics for Eldercare

Deploying social robots in eldercare offers transformative potential, yet it also introduces critical ethical considerations that must be addressed to ensure their responsible use. Key areas of concern include data privacy, emotional dependency, and the need for transparent and secure design. These issues are particularly sensitive when working with vulnerable populations, such as elderly individuals, who may not fully grasp the implications of interacting with autonomous systems. Addressing these concerns is vital to improving trust, safeguarding user dignity, and ensuring that social robots remain beneficial tools for caregiving rather than sources of risk or harm.

Data privacy is one of the most pressing ethical challenges, as social robots often process sensitive user information, such as health data, emotional states, and behavioral patterns. To protect users, adhering to robust legal frameworks such as the General Data Protection Regulation (GDPR) in Europe or similar laws worldwide is essential. Practical measures include collecting only the data necessary for the robot’s functionality, a principle known as data minimization. For example, robots could retain anonymized emotional scores or activity markers rather than storing detailed conversational logs. Encryption during data transmission and storage adds additional protection, making sensitive information inaccessible to unauthorized parties. Anonymization and pseudonymization techniques are also vital, as they prevent personal identifiers from being directly linked to the stored data, even in the event of a security breach. Transparency is equally critical, and users must have full control over their data through consent management systems that clearly outline what data are collected, why they are needed, and how they will be used. Robots should periodically remind users of these privacy settings and provide accessible options for modifying or revoking their consent.

Another significant ethical concern is the risk of emotional dependency. Research by Yuan et al. [58] emphasizes that while these robots can alleviate loneliness, their design must prioritize fostering human connections over replacing them entirely, ensuring a balanced role in caregiving relationships. Even though social robots are designed to provide companionship and emotional support, there is a danger that some users may develop an over-reliance on these systems, potentially diminishing their interactions with family, friends, or caregivers. This dependency can lead to increased isolation over time, contrary to the intended goal of enhancing emotional well-being. To address this, social robots should actively encourage human interactions. For instance, robots could suggest contacting loved ones or remind users of upcoming family visits. Additionally, the design of these robots should carefully limit anthropomorphism. While incorporating some empathetic behaviors, such as a soothing tone or calming gestures, can enhance user experience, over-simulating human-like emotional expressions may blur the lines between machine and human, potentially leading to inappropriate attachment. Periodic reminders of the robot’s role as an assistive device, rather than a substitute for human relationships, are essential.

Transparency and ethical design principles also play crucial roles in ensuring social robots promote trust and empower users [59]. Robots must clearly communicate their capabilities and limitations so that users do not mistakenly attribute undue abilities or intelligence to them. Explainable artificial intelligence (xAI) mechanisms can be integrated to provide context for a robot’s decisions and actions. For example, if a robot suggests a specific physical exercise, it could explain, “I noticed that you haven’t moved much today, so I thought some light stretching might help”. Such interactions help users understand why certain recommendations are made and reinforce the robot’s role as a supportive tool. Furthermore, robots should maintain interaction logs that users or caregivers can review to ensure accountability and transparency. Providing user training materials is equally important, especially for elderly individuals, as these materials can help them navigate the robot’s functions effectively while fostering confidence in its use.

Given the sensitive nature of the data handled by social robots, security must remain a priority. Regular software updates are crucial to address emerging vulnerabilities and ensure the robot complies with evolving regulations. For example, multi-factor authentication (MFA) can provide additional protection for caregivers or family members accessing sensitive information stored on the robot. Third-party audits can further strengthen trust by validating the security and privacy features of the robot through independent evaluations. Such proactive measures reduce the risks of data breaches and reinforce the ethical framework surrounding using social robots.

Beyond technical safeguards, social robots must address broader societal and cultural implications. Designing inclusive and culturally sensitive robots ensures they can adapt to diverse environments and meet the expectations of different populations. For instance, cultural norms may influence preferred communication styles, with some cultures valuing formal interactions and others appreciating more casual and friendly tones. Accessibility is another key consideration. Cost barriers must be addressed to ensure equitable access to these technologies, particularly for low-income populations. Modular designs, where users can purchase only the necessary components, can help reduce production and maintenance costs. Open-source solutions can also play a role by encouraging innovation and reducing development costs, making these systems more affordable and widely available.

## 6. Conclusions and Future Work

This study highlights the transformative role that social robots with cognitive support capabilities can play in enhancing eldercare. The analyzed robots, designed to provide personalized assistance, cater to the unique cognitive and emotional needs of elderly individuals, offering a broad range of services from cognitive exercises to social engagement and assistance with daily tasks. By doing so, they not only promote autonomy but also significantly alleviate the responsibilities placed on caregivers, allowing for a more balanced care environment. The adaptability of these robots to individual user needs enables a truly responsive caregiving solution, one that evolves with the user’s mental and emotional states over time.

While the benefits of these robots are substantial, financial accessibility remains a significant barrier to broader adoption. To address this, several strategies could help reduce costs and expand accessibility. One approach is to develop modular designs, allowing users to purchase only the components they need, which could lower production and maintenance costs while offering tailored functionality. Partnerships with public health systems or insurance providers could also help by subsidizing or partially funding these technologies, particularly for lower-income groups. Open-source software and hardware offer another way to reduce development costs and encourage innovation, enabling broader participation and competition in the market. Additionally, exploring economic feasibility is crucial to understanding the long-term potential of these robots. Cost–benefit analyses could evaluate savings from reduced caregiver workloads and improved quality of life for users, while alternative financing models, such as leasing or subscription-based services, could make adoption more affordable. By addressing these challenges, assistive robots could become accessible to a wider range of users, ensuring their benefits are distributed equitably and effectively integrated into diverse healthcare systems.

Some initiatives have begun exploring solutions to reduce costs and expand accessibility. For instance, the modular robot designs in the Robot Operating System (ROS) open-source platform (Robot Operating System (ROS). https://www.ros.org, accessed on 30 November 2024) have shown promise in reducing production costs by allowing customization and the easy replacement of components. Additionally, collaborations between robotics companies and public health systems, like the European Union’s Horizon 2020 Program, have funded projects to integrate assistive technologies into healthcare services, aiming to make them more affordable for underserved populations: see, for instance, RAMCIP (RAMCIP Project. https://www.fundacioace.com/en/projects/social-research/ramcip---robotic-assistant-for-mci-patients-at-home.html, accessed on 30 November 2024), SPRING (SPRING Project. https://arxiv.org/abs/2404.07560, accessed on 30 November 2024), and GiraffPlus (GiraffPlus Project. https://robots.nu/en/company/giraffplus, accessed on 22 December 2024). Open-source initiatives, such as the OpenAssistive Project (OpenAssistive Project. https://openassistive.org, accessed on 30 November 2024), are also contributing by sharing designs and software for assistive technologies, enabling lower-cost development and broader access. These efforts highlight that, with targeted strategies, assistive robots could become more accessible, offering their benefits to a broader socioeconomic spectrum and easing integration into healthcare systems worldwide.

Future research in the area will focus on improving the robot’s ability to recognize and respond to a broader range of emotions and cognitive states through enhanced machine learning algorithms and multimodal sensor data. Ethical considerations around data privacy, user autonomy, and emotional dependency are also essential areas for further investigation. Future studies should work toward developing a comprehensive ethical framework that ensures these technologies balance effectiveness with ethical boundaries, maintaining users’ dignity and independence. Additionally, expanding user trials across diverse cultural and socioeconomic contexts will provide valuable insights into optimizing robot design and functionality for global eldercare applications.

## Data Availability

The original contributions presented in this study are included in the article. Further inquiries can be directed to the corresponding authors.
